# A technique for continuous bedside monitoring of global cerebral energy state

**DOI:** 10.1186/s40635-016-0077-2

**Published:** 2016-01-20

**Authors:** Rasmus Jakobsen, Troels Halfeld Nielsen, Asger Granfeldt, Palle Toft, Carl-Henrik Nordström

**Affiliations:** Department of Anaesthesia and Intensive care, Odense University Hospital, Sdr. Boulevard 29, 5000 Odense C, Denmark; Department of Neurosurgery, Odense University Hospital, Sdr. Boulevard 29, 5000 Odense C, Denmark; Department of Anesthesiology, Regional Hospital of Randers, Skovlyvej 1, 8930 Randers NØ, Denmark

**Keywords:** Hemorrhagic shock, Microdialysis, Cerebral energy state, Ischemia

## Abstract

**Background:**

Cerebral cytoplasmatic redox state is a sensitive indicator of cerebral oxidative metabolism and is conventionally evaluated from the extracellular lactate/pyruvate (LP) ratio. In the present experimental study of global cerebral ischemia induced by hemorrhagic shock, we investigate whether the LP ratio obtained from microdialysis of cerebral venous blood may be used as a surrogate marker of global cerebral energy state.

**Methods:**

Six female pigs were anesthetized and vital parameters were recorded. Microdialysis catheters were placed in the left parietal lobe, the superior sagittal sinus, and the femoral artery. Hemorrhagic shock was achieved by bleeding the animals to a mean arterial pressure (MAP) of approximately 40 mmHg and kept at a MAP of about 30–40 mmHg for 90 min. The animals were resuscitated with autologous whole blood followed by 3 h of observation.

**Results:**

The LP ratio obtained from the intracerebral and intravenous catheters immediately increased during the period of hemorrhagic shock while the LP ratio in the arterial blood remained close to normal levels. At the end of the experiment, median LP ratio (interquartile range) obtained from the intracerebral, intravenous, and intra-arterial microdialysis catheters were 846 (243–1990), 309 (103–488), and 27 (21–31), respectively. There was a significant difference in the LP ratio obtained from the intravenous location and the intra-arterial location (*P* < 0.001).

**Conclusions:**

During cerebral ischemia induced by severe hemorrhagic shock, intravascular microdialysis of the draining venous blood will exhibit changes of the LP ratio revealing the deterioration of global cerebral oxidative energy metabolism. In neurocritical care, this technique might be used to give information regarding global cerebral energy metabolism in addition to the regional information obtained from intracerebral microdialysis catheters. The technique might also be used to evaluate cerebral energy state in various critical care conditions when insertion of an intracerebral microdialysis catheter may be contraindicated, e.g., resuscitation after cardiac standstill, open-heart surgery, and multi-trauma.

## Background

Intracerebral microdialysis with bedside analysis and display of chemical variables related to cerebral energy metabolism, excitotoxicity, and cell membrane degradation has been available as a clinical routine technique for almost 20 years [1–3]. As the microdialysis probe reflects the biochemistry from a very narrow zone surrounding the dialysis membrane, appropriate positioning and documentation of the position of the catheter in relation to focal lesions is necessary for a correct interpretation of the data obtained [4]. During neurocritical care, e.g., following traumatic brain injury, information regarding global cerebral energy state in addition to the regional information obtained from conventional intracerebral microdialysis would be valuable. Such information would also be of importance during critical care of other severe conditions when cerebral energy metabolism may be jeopardized without focal lesions (e.g., open-heart surgery, resuscitation after cardiac standstill, hemorrhagic or septic shock, toxic states). However, in these conditions, it is for various reasons difficult or impossible to insert intracerebral catheters. It would be of interest to develop an alternative technique that avoids the penetration of cerebral tissue by the microdialysis catheter and still gives continuous bedside information regarding cerebral energy state.

Cerebral cytoplasmatic redox state is a sensitive indicator of cerebral oxidative metabolism and is conventionally evaluated from the extracellular lactate/pyruvate (LP) ratio [5–8]. During insufficient oxygen supply (e.g., arterial hypoxia, ischemia), the LP ratio monitored by intracerebral microdialysis increases instantaneously and if oxygenation is rapidly restored, it returns to a normal or near-normal level [9]. When cerebral oxidative metabolism is compromised by mitochondrial dysfunction, a lasting increase of the LP ratio is observed [8, 10, 11]. Although lactate and pyruvate are water-soluble, they rapidly equilibrate over cell membranes as well as the blood-brain barrier [12–14]. Accordingly, from a theoretical point of view, it might be possible to evaluate changes in global cerebral redox state by monitoring the LP ratio of the cerebral venous outflow.

In the present experimental study, we explore whether cerebral venous LP ratio may be used as a surrogate marker for compromised cerebral oxidative metabolism during hemorrhagic shock. Accordingly, we test the hypothesis that the LP ratio monitored in the cerebral venous outflow reflects the cerebral energy metabolism during compromised conditions and hence is different from the LP ratio monitored in the arterial blood.

## Methods

The study was approved by the National Committee on Animal Research Ethics (2013-15-2934). The depth and duration of hemorrhagic shock necessary for producing cerebral ischemia that caused a compromised energy state and degradation of cell membranes as evaluated from biochemical variables obtained by microdialysis were based on four pilot studies. When the experimental model had been defined, six female pigs approximately 4 months old weighing 42 (35–45) kg were included in the study.

### Anesthesia, mechanical ventilation, and surgical preparation

The porcine model of hemorrhagic shock has been previously described [15]. The animals were fasted overnight with access to ad libitum water. Sedation was achieved with a standard mixture of medetomedin (0.05 mg/Kg), midazolam (0.25 mg/Kg), and atropine (0.25 mg/Kg). Anesthesia was induced with midazolam (0.625 mg/Kg) and ketamine (12.5 mg/Kg) and maintained with infusion of midazolam (5 mg/Kg/h) and fentanyl (50 μg/Kg/h). The animals were intubated and volume-controlled ventilated (Siemens 900 Ventilator; Siemens Elema, Stockholm, Sweden) with a tidal volume of 10 mL/kg and FiO_2_ of 0.30. PaCO_2_ was kept between 4 and 6 kPa and body temperature around normal 38.5 °C.

### Multimodal monitoring

After establishing anesthesia, one sheath was inserted into the carotid artery for blood pressure monitoring and blood gas sampling, while the external jugular vein was cannulated for insertion of a pulmonary artery catheter (CCOmbo; Edwards Lifesciences, Irvine, CA, USA) to monitor cardiac output (CO), core temperature, and central venous pressure (CVP). Arterial blood gases (PaCO_2_, PaO_2_, pH), blood glucose electrolytes, and lactate levels were measured every 30 min (ABL800 FLEX, Radiometer Denmark).

One femoral artery was cannulated for withdrawing and re-infusing blood during the induced hemorrhagic shock. Another sheath was placed in the contralateral femoral artery, and a microdialysis catheter (CMA 70 Bolt, M Dialysis AB, Stockholm, Sweden) was inserted. A small craniotomy was placed in the frontal bone in the midline above the superior sagittal sinus. The sinus was cannulated by a standard 18G peripheral venous catheter, and one microdialysis catheter (CMA 70 Bolt, M Dialysis AB, Stockholm, Sweden) was introduced in the posterior direction and placed in the posterior part of the superior sagittal sinus. The superior sagittal sinus was chosen for analysis of cerebral venous blood due to the anatomic characteristics of the experimental animal. In the pig, most of cerebral blood is drained via paraspinal venous plexa and only a minor part passes into the internal jugular vein [16]. A third microdialysis catheter (CMA 70, M Dialysis AB, Stockholm, Sweden) was inserted 20 mm into the left parietal lobe and one probe for monitoring brain tissue oxygenation (PbtO2) (Licox CC1SB, Integra Neurosciences Ltd., NJ, USA) was introduced 15 mm into the contralateral parietal lobe. A transducer for monitoring intracranial pressure (ICP) (Camino, Integra Neurosciences Ltd., NJ, USA) was placed in the right hemisphere. A bladder catheter was placed for urine collection. All animals were given a baseline dose of 200 U/kg of heparin and supplemented hourly with 100 U/kg for anticoagulation during the hemorrhage period. At the end of experiment, the anesthetized animals were killed with an i.v. injection of sodium pentobarbital 200 mg/mL in concentrated ethanol.

### Experimental protocol

Following a 120-min baseline period allowing animals to stabilize, the following surgery hemorrhagic shock was achieved by bleeding the animals to a pre-defined MAP of approximately 40 mmHg at a rate of 2.15 mL/kg/min over 7 min and then 1.15 mL/kg/min over the remaining period [15]. Animals were kept at a MAP of about 30–40 mmHg by withdrawing or infusing shed blood that was stored in a citrated glucose solution at 37 °C. Following 90 min of hemorrhagic shock, the animals were resuscitated by re-infusing the shed blood at a rate of 120 mL/min until all blood was returned. The pigs were observed for 3 h after hemorrhagic shock. Microdialysis probes were perfused with artificial CSF (M Dialysis AB, Stockholm, Sweden) at a rate of 0.3 μl/min (CMA 106 MD pump, M Dialysis AB, Stockholm, Sweden). The dialysates were collected in microvials and immediately analyzed for glucose, lactate, pyruvate, glutamate, and glycerol every 30 min using an ISCUS^Flex^ analyzer (M Dialysis AB, Stockholm, Sweden). After insertion, all probes were allowed a minimum of 2 h for stabilization.

PbtO_2_ data were collected using the AC3.1 monitor (Integra Neurosciences Ltd.) and recorded every 20 s. All Licox probes were tested against atmospheric air and against each other before insertion and after removal. After insertion, appropriate function was confirmed by an oxygen challenge test.

ICP was monitored continuously and data were collected by a CAM01 monitor (Integra Neurosciences Ltd., NJ, USA) and ICP. Cerebral perfusion pressure (CPP) was calculated as MAP − ICP.

### Statistics

Data are given as median (interquartile range) unless otherwise noted. To test our hypothesis, the time course of the LP ratio in the superior sagital sinus and femoral artery was modeled utilizing a mixed-effects model for repeated measurements with time and location of microdialysis probe as random effects and each animal as fixed effect. The null hypothesis was that no difference in the LP ratio was found between the superior sagital sinus and femoral artery. A *p* value below 0.05 was considered significant. Data analysis was performed in Stata 11.1 statistical software (StataCorp, College Station, TX, USA).

## Results

**Fig. 1:**
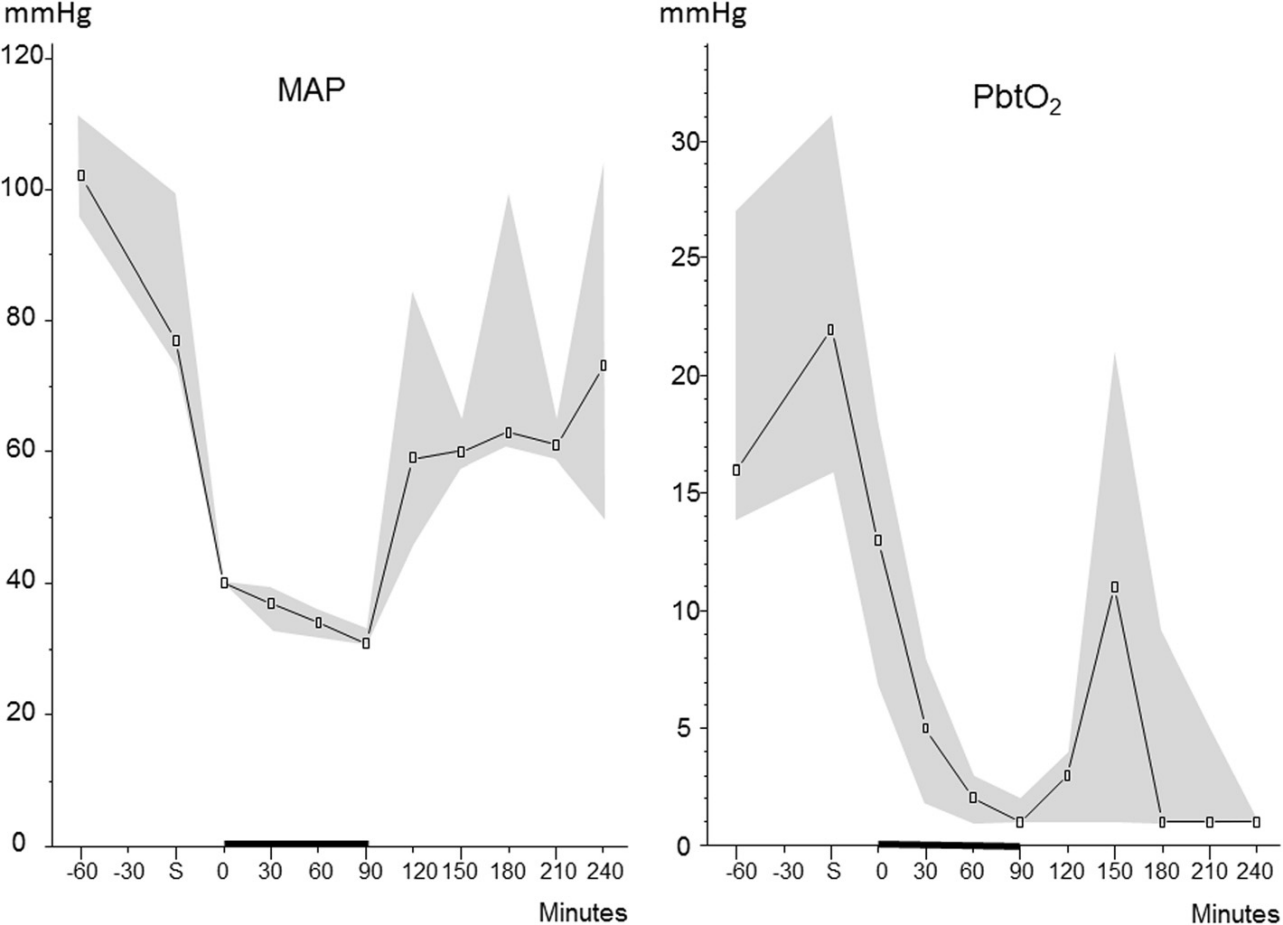
Median (interquartile range) arterial pressure (MAP) and brain tissue oxygen tension (PbtO_2_) in pigs with induced hemorrhagic shock. Note that during the period of hemorrhagic shock, the declining MAP was accompanied by a decrease in PbtO_2_ to a very low level (<5 mmHg). After re-infusion of autologous blood, MAP increased to close to baseline level whereas PbtO_2_ remained very low. *S* indicates the start of bleeding to achieve a MAP of 40 mmHg. The *black bar* on the *x*-axis from 0 to 90 min indicates the shock period before resuscitation was started

Table 1 gives the physiological and general biochemical variables monitored during the experimental period. During the period of hemorrhagic shock, CPP decreased to about 30 mmHg causing a decrease in PbtO_2_ to a very low level (<5 mmHg). After re-infusion of autologous blood, MAP increased close to baseline levels. As ICP continued to increase during the observation period, the CPP remained low (approximately 40 mmHg). The PbtO_2_ remained at very low levels after re-infusion of blood. The relation between MAP and PbtO_2_ is illustrated in Fig. 1. During and after the shock period, PaO_2_, PaCO_2_, and b-glucose remained within normal limits. b-Lactate increased and b-pH decreased during the period of hemorrhagic shock, and both essentially normalized during the observation period. Median blood loss was 1072 mL (964–1498 mL) and median blood loss per kilogram was 31 mL/kg (26–37 mL/kg).

**Table 1:** General physiological and biochemical variables doing hemorrhagic shock

Elapsed time (min)	MAP (mmHg)	ICP (mmHg)	CPP (mmHg)	PbtO_2_ (kPa)	PaO_2_ (kPa)	PaCO_2_ (kPa)
S −60	102 (96–111)	7 (3–11)	95 (90–106)	16 (14–27)	25 (24–27)	6.0 (5.2–6.1)
S	77 (73–99)	8 (2–12)	72 (64–93)	22 (16–31)	24 (23–25)	5.7 (5.4–6.0)
0	40 (40–40)	8 (6–8)	32 (28–34)	13 (7–18)	24 (23–25)	5.6 (5.5–5.6)
30	37 (33–39)	5 (1–9)	29 (29–31)	5 (2–8)	25 (25–26)	5.9 (5.2–6.5)
60	34 (32–36)	5 (0–8)	29 (28–31)	2 (1–3)	24 (24–25)	5.7 (5.2–6.0)
90	31 (31–33)	6 (0–13)	32 (25–43)	1 (1–2)	26 (26–28)	5.0 (4.6–7.2)
120	59 (46–84)	17 (13–22)	51 (38–62)	3 (1–4)	25 (24–26)	5.6 (4.5–6.3)
150	60 (58–65)	19 (14–28)	46 (46–47)	11 (1–21)	22 (22–24)	6.5 (5.6–7.3)
180	63 (61–99)	27 (20–34)	42 (41–65)	1 (1–9)	23 (22–23)	5.1 (4.6–5.7)
210	61 (59–65)	23 (21–31)	42 (40–42)	1 (1–5)	22 (21–22)	5.4 (4.8–5.6)
240	73 (50–104)	29 (24–39)	47 (39–49)	1 (1–1)	22 (21–23)	6.1 (5.4–6.4)
Elapsed time (min)	b-Hemoglobin (mM/L)	HR (bpm)	b-Glucose (mM/L)	b-Lactate (mM/L)	b-pH	Diuresis (mL)
S −60	5.7 (5.3–5.8)	78 (75–84)	6.8 (6.4–7.1)	1.5 (1.3–1.7)	7.43 (7.40–7.47)	40 (4–106)
S	5.6 (5.2–5.9	90 (78–93)	7.2 (6.8–7.7)	1.2 (1.0–1.4)	7.45 (7.43–7.46)	115 (82–160
0	4.9 (4.7–4.9)	135 (107–138)	9.7 (8.9–9.8)	2.5 (2.0–3.0)	7.41 (7.41–7.43)	12 (0–23)
30	4.6 (4.5–4.7)	116 (111–146)	8.6 (7.7–14.4)	5.3 (3.7–7.4)	7.34 (7.32–7.38)	4 (3–5)
60	5.2 (4.9–5.5)	166 (129–191	7.9 (6.2–13.1)	7.2 (4.7–11.1)	7.30 (7.25–7.41)	2 (1–5)
90	5.2 (5.2–5.4)	125 (114–168)	7.0 (4.8–9.2)	10.3 (6.0–10.3)	7.20 (7.18–7.31)	3 (2–10)
120	5.1 (4.6–5.6)	98 (93–116)	6.2 (5.2–7.0)	5.5 (4.4–6.5)	7.29 (7.21–7.34)	16 (5–31)
150	5.2 (5.1–5.6)	101 95–120)	6.4 (4.9–7.6)	4.8 (3.6–6.9)	7.20 (7.19–7.24)	20 (17–25)
180	5.5 (5.2–5.8)	110 (103–113)	6.5 (5.7–8.5)	2.5 (2.1–3.7)	7.43 (7.33–7.50)	70 (25–70)
210	5.5 (5.3–5.7)	122 (88–128)	4.5 (3.9–5.4)	2.1 (1.7–3.3)	7.43 (7.39–7.43)	45 (5–45)
240	5.8 5.7–5.8)	17 (105–121)	5.5 (4.6–6.1)	2.0 (1.2–3.0)	7.36 (7.33–7.47)	18 (5–100)

Data are expressed as median levels (interquartile range). S indicates the start of bleeding to achieve a MAP of 40 mmHg

*MAP* mean arterial pressure, *ICP* intracranial pressure, *CPP* cerebral perfusion pressure, *PbtO*_*2*_ brain tissue oxygenation.

Table 2 gives the biochemical variables obtained from microdialysis catheters positioned in the cerebral hemisphere, in the superior sagittal sinus, and in the femoral artery, respectively. Before the induction of hemorrhagic shock, LP ratios were similar (11–18) in all three catheter positions and within normal limits [17]. In the cerebral hemisphere, hemorrhagic shock caused a marked increase of the LP ratio that increased further after re-infusion of blood. The increase of the LP ratio was caused by a pronounced increase in lactate simultaneously with a pronounced and lasting decrease in pyruvate. Intracerebral glucose decreased and remained at a very low level during the whole study period (<1 mmol/L). Extracellular intracerebral glutamate and glycerol increased during the shock period and remained at very high levels after re-infusion of blood.

**Table 2:** Biochemical variables obtained from microdialysis

Elapsed time (min)	LP ratio			Lactate (mM/L)			Pyruvate (μM/L)		
	Hemisphere	Sagittal sinus	Femoral	Hemisphere	Sagittal sinus	Femoral	Hemisphere	Sagittal sinus	Femoral
S −60	15 (14–18)	13 (9–16)	11 (11–19)	20.7 (10.7–30.0)	20.1 (10.7–20.3)	10.6 (10.1–1.9)	131 (107–178)	111 (106–123)	141 (99–160)
S −30	13 (11–16)	13 (6–16)	12 (10–17)	2.9 (2.4–3.5)	2.2 (2.2–2.2)	2 (1.7–2.6)	191 (151–205)	200 (193–371)	166 (161–175)
S	15 (11–18)	14 (8–26)	13 (10–21)	2.4 (2.1–3.1)	3.4 (2.6–5.2)	2.2 (1.6–3.4)	185 (153–210)	220 (161–388)	137 (131–153)
0	20 (17–41)	45 (32–46)	33 (30–36)	7.6 (7.2–8.0)	11.1 (10.5–11.7)	3 (2.3–4.0)	110 (107–149)	178 (144–208)	181 (129–193)
30	60 (27–117)	36 (23–63)	27 (26–27)	10.3 (7.8–11.0)	6.8 (4.6–12.1)	3.8 (3.7–4.4)	143 (92–163)	222 (147–230)	181 (111–187)
60	169 (45–418)	85 (36–112)	28 (26–35)	14.6 (11.8–17.2)	9 (6.6–13.2)	5.5 (5.2–6.8)	85 (43–137)	219 (128–272)	214 (144–286)
90	351 (105–774)	78 (42–117)	29 (25–33)	14.9 (10.2–18.8)	10.2 (7.0–13.6)	7.8 (4.2–9.3)	36 (21–49)	175 (111–208)	312 (256–348)
120	704 (97–1454)	94 (63–135)	29 (25–35)	12.9 (10.2–17.2)	9.7 (8.0–12.0)	9.7 (4.0–12.9)	28 (9–64)	129 (68–232)	305 (191–348)
150	879 (115–1238)	123 (36–332)	34 (24–37)	11.2 (10.6–15.9)	8.9 (8.0–13.8)	6.4 (3.0–10.9)	24 (9–210)	80 (29–269)	266 (162–308)
180	724 (264–2479)	150 (63–308)	30 (22–34)	12.9 (9.7–14.6)	8.8 (6.8–13.1)	7 (3.5–9.8)	38 (6–158)	90 (34–168)	215 157–274)
210	846 (243–1990)	309 (103–488)	27 (21–31)	13.4 (11.2–13.8)	9.6 (8.0–14.1)	4.4 (3.1–7.7)	8 (2–94)	81 (15–150)	192 (171–286)
Elapsed time (h)	Glucose (mM/L)			Glutamate (μM/L)			Glycerol (μM/L)		
	Hemisphere	Sagittal sinus	Femoral	Hemisphere	Sagittal sinus	Femoral	Hemisphere	Sagittal sinus	Femoral
S −01:00	2.4 (1.2–3.7)	3 (1.3–4.0)	5.2 (3.1–5.3)	5 (5–13)	130 (75–168)	183 (168–200)	27 (15–50)	31 (17–57)	34 (28–54)
S −00:30	2.9 (2.4–3.7)	2.9 (1.1–3.7)	4.1 (2.4–7.1)	8 (3–11)	162 (121–177)	199 (159–209)	26 (14–55)	28 (24–63)	34 (26–35)
S	3.6 (2.7–4.1)	1.7 (1.3–3.8)	3.7 (2.1–6.5	6 (4–7)	174 (104–204)	180 (148–209)	23 (13–41)	31 (23–55)	21 (20–29)
0	1.7 (1.5–1.9)	–	1.2 (0.7–1.6)	–	87 (50–121)	202 (169–221)	40 (40–40)	74 (49–100)	12 (11–14)
30	1.9 (1.2–2.4)	1.3 (0.6–4.2)	6.9 (1.3–9.2)	19 (4–55)	158 (84–159)	185 (163–206)	56 (32–108)	53 (37–112)	21 (8–32)
60	1.0 (0.7–1.4)	2 (0.02–2.2)	3.8 (1.1–8.2)	68 (36–101)	156 (132–204)	181 (158–204)	180 (133–201)	96 (53–159)	33 (14–117)
90	0.4 (0.3–0.5)	0.6 (0.02–1.5)	5.4 (1.7–7.6)	119 (119–119)	225 (204–248)	172 (149–195)	329 (254–337)	120 (102–190)	89 (29–486)
120	0.2 (1.0–0.3)	0.3 (0.04–0.7)	3.4 (1.2–7.3)	245 (199–291)	209 (209–209)	192 (152–198)	415 (318–455)	192 (123–258)	83 (23–671)
150	0.1 (0.1–1.0)	0.1 (0.02–0.5)	3.1 (0.5–5.6)	327 (231–329)	199 (152–259)	153 (107–177)	403 (377–548)	222 (151–286)	76 (19–621)
180	0.2 (0.1–1.3)	0.1 (0.02–0.2)	1.8 (0.5–5.9)	335 (289–367)	198 (133–256)	173 (139–180)	382 (333–547)	213 (191–230)	71 (18–314)
210	0.3 (0.1–0.9)	0.02 (0.02–0.7)	5.5 (0.9–8.1)	311 (232–322)	257 (227–287)	186 (184–188)	522 (474–612)	321 (268–331)	101 (26–182)

Data are expressed as median (interquartile range). S indicates the start of bleeding to achieve a MAP of 40 mmHg

*LP* lactate/pyruvate ratio

**Fig. 2:**
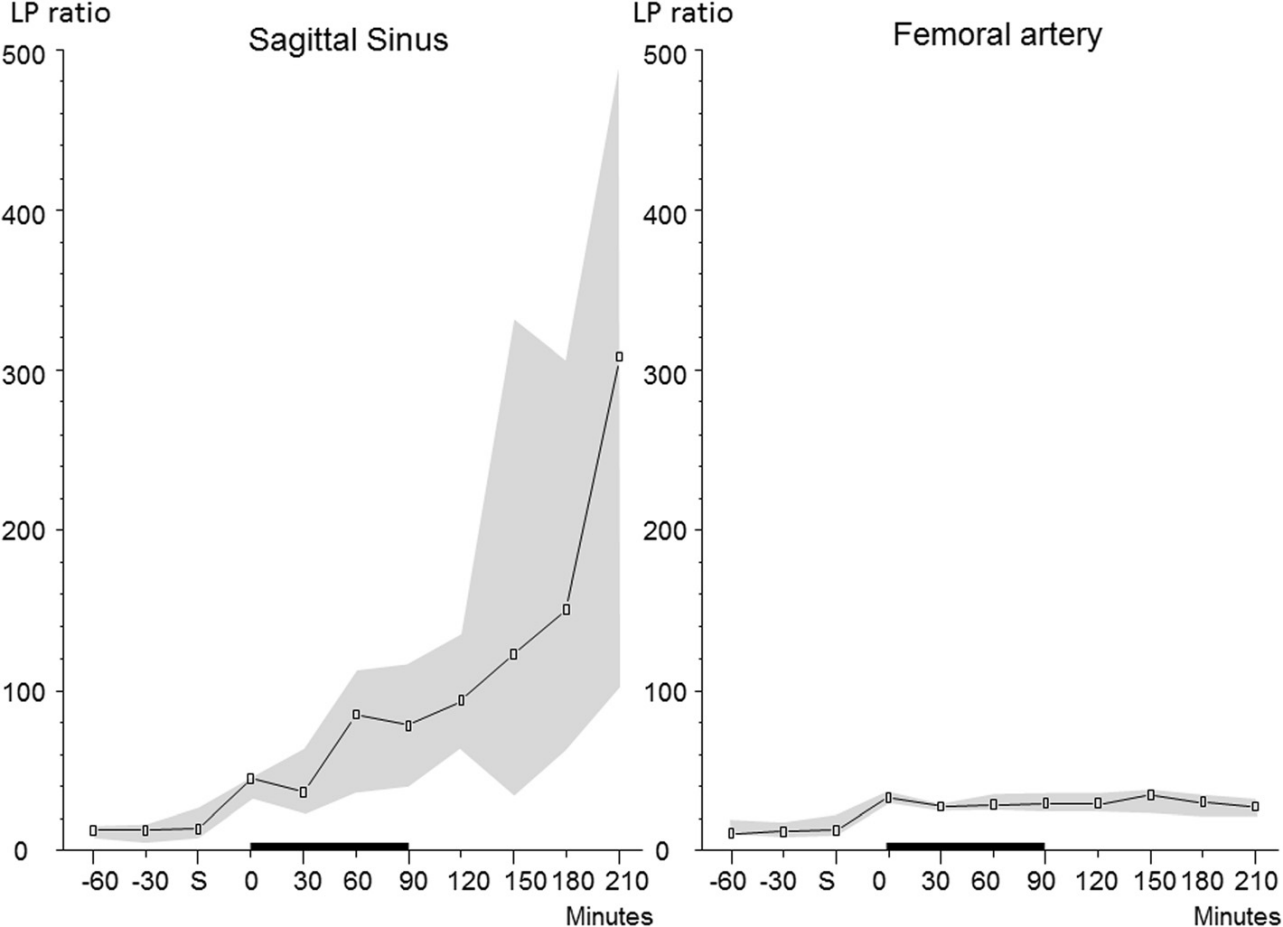
Logarithmic illustration of the LP ratios (median (interquartile range)) in the sagittal sinus and femoral artery during hemorrhagic shock in pigs. The increase in the LP ratio in the sagittal sinus was significantly higher (*p* < 0.001) than in the arterial blood. *S* indicates the start of bleeding to achieve a MAP of 40 mmHg. The *black bar* on the *x*-axis from 0 to 90 min indicates the shock period before resuscitation was started

**Fig. 3:**
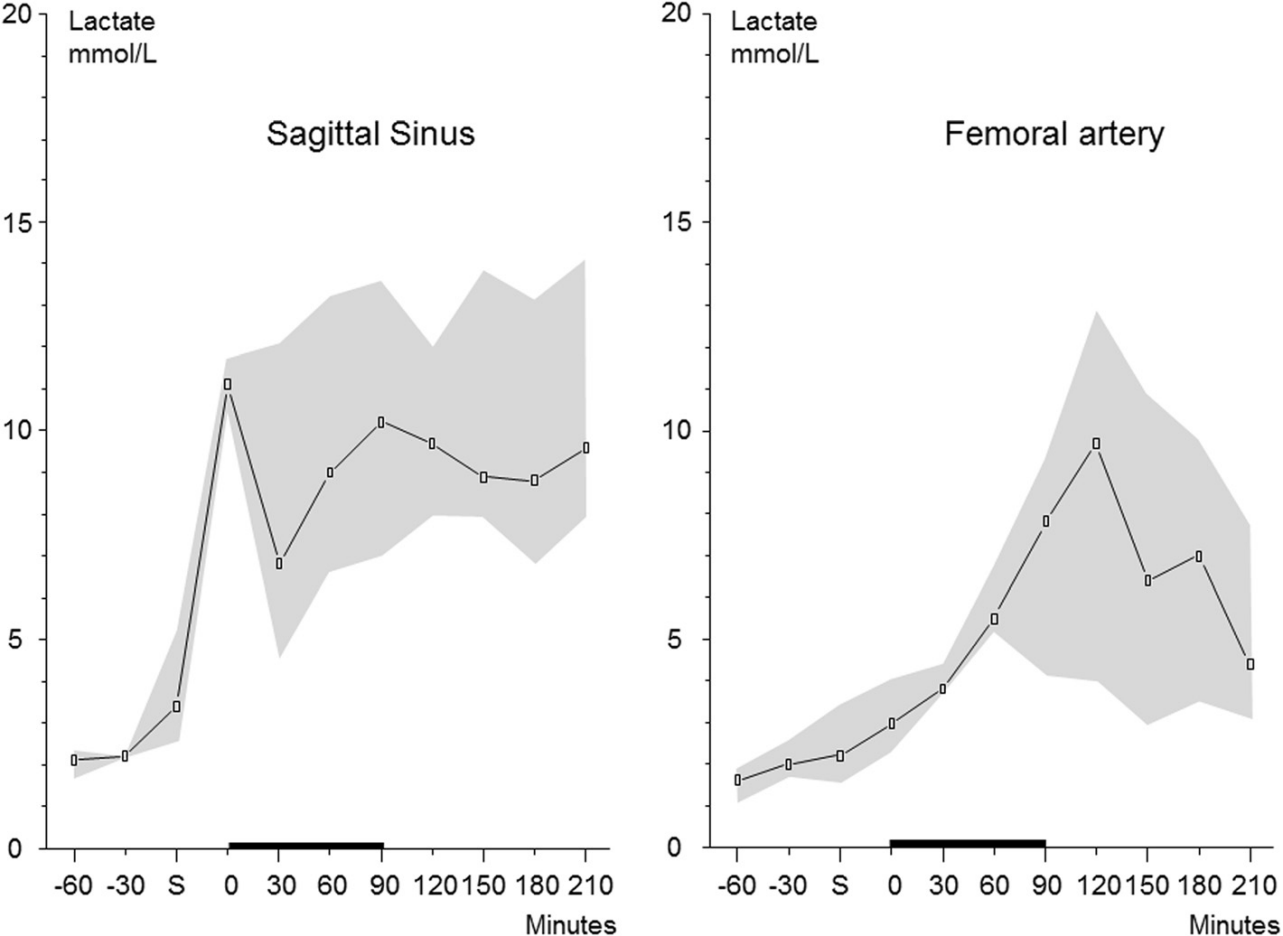
Microdialysis levels of lactate (median (interquartile range)) in the sagittal sinus and femoral artery doing hemorrhagic shock in pigs. Note that lactate levels increases in both compartments during shock. After re-infusion of blood, lactate levels in the sagittal sinus remain at a high level throughout the monitoring period. In contrast, the lactate levels in the femoral artery decline to a near-normal level. *S* indicates the start of bleeding to achieve a MAP of 40 mmHg

In the superior sagittal sinus, the LP ratio increased during the shock period and continued to increase to a very high level (100–500) after blood transfusion. In the femoral artery, the shock period was associated with a modest increase of the LP ratio but remained close to the upper reference level (30) in normal cerebral tissue [17]. In the superior sagittal sinus, the pronounced increase in the LP ratio was caused by a marked increase in lactate simultaneously with a marked decreased in pyruvate. In the femoral artery, the increase in lactate concentration was accompanied by a simultaneous increase in pyruvate limiting the increase in the LP ratio. The time courses of the changes in the LP ratios in the sagittal sinus and femoral artery are shown in Fig. 2. Figure 3 shows the simultaneous changes in lactate concentration in the two compartments. The difference in the LP ratio between the superior sagittal sinus and femoral artery was significant (*p* < 0.001).

After induction of hemorrhagic shock, glucose concentration decreased to extremely low levels in the superior sagittal sinus that lasted during the whole study period. In the femoral artery, glucose remained relatively constant. Already before induction of hemorrhagic shock (control conditions), glutamate concentration was very high in the superior sagittal sinus as well as in the femoral artery (100–200 μmol/L). In both positions, glutamate remained essentially unchanged during the study period. Glycerol concentration increased in the superior sagittal sinus as well as in the femoral artery during the shock period and continued to increase after re-infusion of blood.

## Discussion

Cerebral cytoplasmatic redox state is primarily determined by mitochondrial oxidative metabolism [3–7]. It is conventionally described by the ratio between the cytoplasmatic levels of lactate and pyruvate and is expressed in the ratio between lactate and pyruvate, the LP ratio.

Lactate and pyruvate are water-soluble. However, due to monocarboxylate transporters (MCTs), they equilibrate rapidly across cellular membranes. MCTs are proton-linked membrane carriers involved in the transport of various monocarboxylates such as lactate, pyruvate, and ketone bodies [12–14, 18]. They are present in all tissues. Out of the total family of 14 members, three isoforms (MCT1, MCT2, MCT4) have been described in the brain [19]. The driving forces for the transport of the monocarboxylates are obtained from the concentration differences over the cellular membranes. The transport is consequently characterized as facilitated diffusion [20]. The MCTs appear to be very effective in transporting lactate and pyruvate. After induction of cerebral ischemia, the intracerebral microdialysis probe will detect an increase in the LP ratio instantaneously and if the circulation is rapidly restored, the LP ratio quickly returns to a normal or near-normal level [9]. Based on these conditions, the present experimental study was designed to explore whether the equilibration of lactate and pyruvate across the blood-brain barrier (BBB) was rapid enough to permit the LP ratio of the draining venous blood to be used as a surrogate marker for global cerebral redox state.

After induction of hemorrhagic shock, the intracerebral LP ratio rapidly increased to a very high level. The increase was due to a marked increase in lactate concentration simultaneously with a pronounced decrease in pyruvate (Table 2, Fig. 2). This metabolic pattern is characteristic of ischemia (i.e., a simultaneous decrease in tissue oxygenation supply and substrate/glucose) [8–10]. After re-infusion of blood, the increase in lactate and the LP ratio increased further while pyruvate concentration continued to decrease. Tissue biochemical analysis thus revealed an insufficient supply of oxygen as well as substrate during the induced hemorrhagic shock that continued after blood transfusion and normalization of MAP.

In the superior sagittal sinus, the LP ratio exhibited a parallel though less pronounced pattern (Table 2, Fig. 2). Like in the cerebral tissue, the microdialysis catheter in the sagittal sinus continued to reveal a markedly elevated LP ratio after re-infusion of blood and the pyruvate concentration decreased to a very low level. In the femoral artery, a modest increase in the LP ratio and a moderate increase in the pyruvate level were obtained during and after the induced hemorrhagic shock. During the whole experimental period, the LP ratio of the femoral arterial blood remained close to the upper reference level (≤30) of normal cerebral tissue [17]. The difference between the level of LP ratio in the superior sagittal sinus and femoral artery after induction of hemorrhagic shock was highly significant, and our null hypothesis was rejected. Accordingly, we conclude that the LP ratio monitored in cerebral venous blood reflected the pronounced global intracerebral redox shift and was not caused by affected energy metabolism in extracranial tissues.

As shown in Table 2 and Fig. 3, the lactate concentration in the superior sagittal sinus increased during and after the period of shock. However, as arterial lactate level also increased markedly, intravenous lactate monitoring alone cannot be used as a marker of compromised cerebral energy metabolism.

### PbtO_2_ and intracerebral glucose

PaO_2_ and b-glucose levels were kept relatively constant during the experimental period (Table 1). In spite of this fact, PbtO_2_ and intracerebral glucose decreased to very low levels during the induced hypotensive shock and remained very low after re-infusion of blood (Table 1, Fig. 1). This pattern is compatible with that observed during cerebral ischemia and corroborates the intracerebral microdialysis findings (Table 2). Accordingly, although MAP returned to close to the initial level after blood re-infusion (Table 1), cerebral perfusion was obviously not sufficient for restoring energy metabolism. The finding is probably explained by the fact that a progressive increase in ICP caused a decrease of CPP to a low level (40 mmHg; Table 1). The reason for the progressive increase in ICP is probably because of a global postischemic cytotoxic edema and leakage of the blood-brain barrier. Due to insufficient perfusion, arterial blood glucose was virtually completely extracted which resulted in a very low glucose level in the sagittal sinus (Table 2).

### Glutamate and glycerol

During clinical intracerebral microdialysis use, an increase in glutamate concentration is generally interpreted as insufficient astrocytic uptake of released glutamate due to energy failure [21, 22]. In the present study, intracerebral glutamate increased markedly during the hypotensive shock period and did not return to normal level after blood re-infusion (Table 2). Thus, the observed changes in intracerebral glutamate are in accordance with the interpretation above: hypotensive shock caused cerebral ischemia and energy failure that did not recover after blood transfusion.

The normal blood-brain barrier is not permeable to glutamate [21]. Under normal conditions, interstitial cerebral concentration is approximately 2 μmol/L while blood concentration is 100–200 μmol/L. Accordingly, glutamate level obtained in cerebral venous blood does not reflect the intracerebral level. In the present study, the high concentration of glutamate obtained before the start of the experiment (100–200 μmol/L; Table 2) documents that the microdialysis catheter was actually positioned in the superior sagittal sinus in each experimental animals.

Intracerebral glycerol measured by microdialysis is conventionally used as a marker of degradation of cellular membranes into free fatty acids and glycerol [23, 24]. In the present experimental situation, intracerebral glycerol increased to a very high level during hypotensive shock and remained at this high level after transfusion (Table 2). The finding supports the interpretation that induced hemorrhagic shock to MAP 30 mmHg for 90 min caused cerebral energy failure and decomposition of cellular elements. However, increase in glycerol in cerebral venous blood (Table 2) does not necessarily result from degradation of cerebral cellular elements. The intact BBB has a very low permeability for glycerol [25]. In many extracerebral tissues, triglycerides are important cellular components. During stress and increased sympathetic tonus, triglycerides are degraded, which is reflected in fat tissue and in the blood as an increase of free fatty acids and glycerol [26]. Accordingly, an increase in glycerol concentration was in the present experimental situation also obtained in the femoral arterial blood (Table 2).

### Clinical relevance of the experimental model

The study indicates that it is possible to evaluate global cerebral energy state by simultaneous monitoring of the redox state (LP ratio) in a cerebral vein. Under clinical conditions, this could be performed by placing the venous microdialysis catheter in the internal jugular vein close to the jugular bulb. In this way, it might be possible to continuously evaluate cerebral energy state bedside without inserting an intracerebral probe. This technique would be valuable in various serious conditions in need of critical care.

After cardiac standstill and resuscitation, the possibilities of evaluating cerebral damage and prognosis are still limited [27–30]. In these patients, a bedside continuous technique might also be used to monitor the effects of various therapies (e.g., hypothermia). For this purpose, intracerebral microdialysis has been used in a few selected cases [31] but it is unlikely that this invasive technique will be used in clinical routine. In patients subjected to open-heart surgery with or without cardiopulmonary bypass, minor cerebral complications appear to be frequent [32–36]. In these patients, analysis of lactate concentration from a microdialysis catheter positioned in a central vein has been proposed [37]. Although this technique was shown to give reliable information regarding global venous lactate level, it will not give specific information regarding cerebral energy metabolism. In patients with hepatic failure (HF) leading to cerebral symptoms and coma, intracerebral microdialysis has shown that an increase in tissue LP ratio is correlated to increases in tissue glutamine and hypoxanthine [38, 39] and energy failure appears to be an important pathogenetic component of both acute and chronic HF and a potential target for therapy [40].

The technique of evaluating global cerebral energy/redox state from the LP ratio obtained from a microdialysis catheter positioned in the internal jugular vein might give important information in a multitude of severe clinical conditions when direct measurements of tissue biochemistry is difficult or impossible. However, it should be recalled that evaluation of the LP ratio in the draining vein will not give quantitative, correct information regarding cerebral extracellular LP ratio (Table 2). This is of limited importance. Under clinical routine conditions, an upper normal level for the LP ratio is utilized (usually 30 or 40) and the exact level of the LP ratio is often of secondary importance [11, 41]. During intracerebral microdialysis, the LP ratio and the concentration of pyruvate have also been used to differentiate between ischemia and mitochondrial dysfunction [8, 10, 11]. This kind of detailed analysis and interpretation may not be possible when the LP ratio is monitored in cerebral venous blood.

During neurocritical care, the cerebral interstitial levels of glutamate and glycerol are used as indicators of insufficient energy production and cellular degradation. However, if the microdialysis catheter is positioned in cerebral venous blood, these interpretations are not valid for reasons given above.

### Limitations

In experimental hemorrhagic shock, it has been described that, in contrast to the systemic macrocirculation, cerebral microcirculation may be remarkably well preserved [42]. Though the implications of this finding have been questioned, it is still an open question when and to what degree cerebral energy metabolism is compromised during hemorrhagic shock under clinical conditions [43]. In a recent experimental study utilizing multimodal monitoring with simultaneous imaging of cerebral hemodynamics and NADH signals, the authors demonstrated the temporal relationship between compromised microcirculation and compromised oxidative metabolism [44]. In this model of severe hemorrhagic shock, the oxidative metabolism was not restored after re-transfusion of the extracted blood volume. From that study and the present data, it appears that during severe hemorrhagic shock cerebral energy metabolism is severely compromised exhibiting a biochemical pattern typical of ischemia. Further, if hypotension is protracted and severe enough, cerebral energy metabolism may not be restored after transfusion. In the present experimental study, the biochemical pattern and the progressive increase of ICP indicated permanent cerebral lesions. The present experimental model was chosen because it creates reproducible severe global cerebral ischemia. However, the chosen hypotensive level of MAP around 35 mmHg is somewhat lower that the recommendations by the European Society for Intensive Care Medicine expert panel [45]. It is therefore important to stress that the present study cannot be used to determine the optimal level of MAP after hemorrhagic shock. The purpose of the present study was solely to establish a technique for “non-cranial” invasive monitoring of cerebral energy state. As shown in Table 2, there is a quantitative discrepancy in the LP ratio between the cerebral microdialysis probe and the one placed in the sinus. This suggests a “washout” effect. The degree of metabolic derangement in the present study was severe. In a clinical setting, e.g., after cardiac standstill, a less pronounced metabolic derangement will be expected. Accordingly, it might not be possible to detect minor metabolic derangements in the venous jugular bulb due to the washout effect. Future clinical studies are needed to determine this.

## Conclusions

This experimental study documents that during protracted (≈90 min) and pronounced (MAP≈35 mmHg) hemorrhagic shock, cerebral energy metabolism was severely compromised and exhibited a biochemical pattern typical of ischemia, energy failure, and cellular degradation. After re-transfusion, this pattern was even more pronounced indicating irreversible tissue damage. From intravascular microdialysis in the superior sagittal sinus, it is possible to achieve semi-quantitative information of the cerebral cytoplasmatic redox state (LP ratio) that can be separated from the biochemical alterations in extracranial tissues. Accordingly, it is possible to monitor global cerebral energy state continuously with a strictly extracerebral technique. This technique might be valuable during critical care of various severe conditions where cerebral energy metabolism may be compromised, e.g., resuscitation after cardiac standstill, open-heart surgery, multi-trauma, hemorrhagic or septic shock, and hepatic coma. Studies are in progress in these clinical conditions and will reveal whether the proposed technique for evaluating global cerebral energy state will be added to the methods to measure and monitor brain function that have evolved in recent years [46].
